# Respiratory syncytial virus-associated mortality in a healthy 3-year-old child: a case report

**DOI:** 10.1186/s12887-019-1847-2

**Published:** 2019-11-27

**Authors:** A. Gavotto, A. Ousselin, O. Pidoux, P. Cathala, V. Costes-Martineau, B. Rivière, J. L. Pasquié, P. Amedro, C. Rambaud, G. Cambonie

**Affiliations:** 1 Pediatric and Congenital Cardiology Department, Arnaud de Villeneuve Hospital, Montpellier University Hospital Center, Montpellier, France; 2 CNRS UMR 9214, INSERM U1046, University of Montpellier, Montpellier, France; 3 Department of Neonatal Medicine and Pediatric Intensive Care, Arnaud de Villeneuve Hospital, Montpellier University Hospital Center, 371 Avenue du Doyen Gaston Giraud, 34295 Montpellier Cedex 5, France; 4 Department of Forensic Medicine, Lapeyronie Hospital, Montpellier University Hospital Center, Montpellier, France; 5 Department of Pathology, Lapeyronie Hospital, Montpellier University Hospital Center, Montpellier, France; 6 Department of Cardiology, Arnaud de Villeneuve Hospital, Montpellier University Hospital Center, Montpellier, France; 7 Department of Pathology and Forensic Medicine, Raymond Poincaré Hospital, Garches University Hospital, Garches, France

**Keywords:** Sudden unexpected death in childhood, Respiratory syncytial virus, Myoepicarditis, Cardioneuropathy, Myosin light chain 2

## Abstract

**Background:**

Respiratory syncytial virus (RSV) is the most frequently identified pathogen in children with acute lower respiratory tract infection. Fatal cases have mainly been reported during the first 6 months of life or in the presence of comorbidity.

**Case presentation:**

A 47-month-old girl was admitted to the pediatric intensive care unit following sudden cardiopulmonary arrest occurring at home. The electrocardiogram showed cardiac asystole, which was refractory to prolonged resuscitation efforts. Postmortem analyses detected RSV by polymerase chain reaction in an abundant, exudative pericardial effusion. Histopathological examination was consistent with viral myoepicarditis, including an inflammatory process affecting cardiac nerves and ganglia. Molecular analysis of sudden unexplained death genes identified a heterozygous mutation in myosin light chain 2, which was also found in two other healthy members of the family. Additional expert interpretation of the cardiac histology confirmed the absence of arrhythmogenic right ventricular dysplasia or hypertrophic cardiomyopathy.

**Conclusions:**

RSV-related sudden death in a normally developing child of this age is exceptional. This case highlights the risk of extrapulmonary manifestations associated with this infection, particularly arrhythmia induced by inflammatory phenomena affecting the cardiac autonomic nervous system. The role of the mutation in this context is uncertain, and it is therefore necessary to continue to assess how this pathogenic variant contributes to unexpected sudden death in childhood.

## Background

Sudden unexpected death in childhood (SUDC) “encompasses all cases in which there is death (or collapse leading to death) of a child, which would not have been reasonably expected to occur 24 hours previously and in whom no pre-existing medical cause of death is apparent” [1]. As in cases of sudden unexpected death in infancy (SUDI), the main goal of the management protocol is to determine the cause of death, attend to the family with appropriate responses to their questions or doubts about the circumstances and cause of the death, and alert the judicial authority if necessary [2]. In addition to the individual dimension of care, public health and disease prevention are broader dimensions aimed at preventing such deaths in the future. The implementation of national registers has recently been proposed to standardize the investigations, develop research and partnerships in this field, and optimize the support and medical evaluation of families [3–5].

According to recent reviews, infection is the most common cause of non-traumatic SUDC, accounting for 36–68% of explained deaths [6, 7]. Evidence for the role of undiagnosed inherited cardiac conditions is also increasing [8]. In a nationwide autopsy study in the Netherlands, cardiovascular disease was the pathological substrate of SUDC in nearly 25% of the cases. In another quarter of the cases, the autopsy found no or only minor cardiac stuctural abnormalities, suggesting the possibility of genetic arrhythmias in a substantial number of patients [9]. These results underscored the importance of cardiovascular examination and genetic testing of SUDC victims at autopsy, as well as their relatives.

We here present a case of SUDC in a girl of nearly 4 years old, whose postmortem analyses revealed the association of viral myoepicarditis at autopsy and a genetic variant in a cardiac gene at molecular autopsy.

## Case presentation

We admitted a child of 3 years and 11 months to the pediatric intensive care unit (PICU) of Montpellier University Hospital following a sudden collapse at home.

The event occurred at about 1 pm, approximately 5 min after the child woke from a nap showing sudden eye rolling and loss of consciousness, but no abnormal movements. The father made the decision to transport her in his vehicle to the nearest medical center. Upon arrival 15 min later, the child was lifeless with cardiac asystole on electrocardiogram (ECG) and the medical team began cardiopulmonary resuscitation (CPR).

At admission, the child was pale and cold and showed no hematoma, purpura, rash or wound. Abundant digestive hemorrhage of black blood was aspirated by a gastric tube. Expired CO_2_ was very low, 20 mmHg, indicating prolonged low blood flow. ECG monitoring showed persistent asystole. After discussion with the referral service, there was no indication for circulatory extracorporeal membrane oxygenation. Resuscitation was stopped and death declared after 80 min of CPR performed by health professionals.

The parents were immediately interviewed and they asked for all exams, including an autopsy, to identify the cause of their child’s death. The forensic pathologist was contacted and, after being fully informed of all events prior to the child’s death, decided that an autopsy should be carried out, for both legal and medical purposes.

This girl was the first child of non-consanguineous Caucasian parents, born full-term with low weight for gestational age (2330 g at 39 weeks of gestation). She was without significant health problems and her vaccinations were up to date on her health record. The child had had gastroenteritis 3 weeks earlier. Two days before the event, she had a fever associated with fatigue and abdominal pain in the right iliac fossa. For this reason, she was kept at home and was examined by the general practitioner, who found no worrisome signs. Treatment with trimebutine maleate and domperidone was prescribed. The previous night, the father noted a small amount of vomiting and more frequent liquid intake. In the morning, she woke at 7 am with an occipital headache relieved with paracetamol. When she woke from her nap, she spoke correctly and had perioral cyanosis without any other sign of respiratory distress.

As for her family, her father had convulsions in childhood, from 6 months to 12 years, and was treated intermittently with diazepam. Her maternal great-grandmother died suddenly at the age of 25–35 years from a ruptured intracranial aneurysm.

Immediately after death, transthoracic puncture in the cardiac area was performed to collect blood. The puncture, however, brought back 10 ml of yellow serous fluid, which prompted an echocardiography that revealed a large pericardial effusion. The analysis of the pericardial fluid was indicative of an exudate (albumin 46 g/L, protein 66.4 g/L, LDH 344 IU/L), with 440 cells/mm^3^ (73% granulocytes, 24% lymphocytes). Direct examination by Gram staining and bacterial culture was negative. The search for a panel of respiratory viruses by real-time polymerase chain reaction (PCR) was positive for respiratory syncytial virus (RSV).

Bacterial cultures of urine, cerebrospinal fluid (CSF) and blood were negative, and stool culture found neither fungal nor specific pathogenic bacteria, including clostridium botulinum. Bordetella pertussis and parapertussis were not found in nasal swabs. No other bacterial or viral analysis was performed on nasal specimens. The search for viruses in CSF, stool, and blood was also negative. Laboratory tests found normal concentrations for hemoglobin, C-reactive protein, and procalcitonin, while leukocyte (19.4 10^9^/L) and platelet (448 10^9^/L) counts were slightly elevated. The serum levels of immunoglobulins were normal for the age. Chromatography found no abnormal peak suggestive of aminoacidopathy, and the acylcarnitine profile was normal. No psychotropic or narcotic drugs were detected in the blood. Serum paracetamol concentration was within the therapeutic range (11.6 mg/L) but domperidone was undetectable. The carboxyhemoglobin level measured in the blood sample at admission was 0%. Radiography of the entire skeleton found no significant abnormality, including no recent or old fracture.

The autopsy was performed 48 h after death. External examination found normal development: weight 14.4 kg; height 100 cm. There was no morphological abnormality and no lesion suggestive of maltreatment or trauma. The internal examination found no malformation or visceral malposition. There was nonspecific polyvisceral congestion, as well as global cerebral edema without hemorrhage or mass effect, which could be attributed to the prolonged resuscitation. Several centimetric and infracentimetric flexible lymphadenopathies were observed at the cervical and mesenteric levels, which may be trivial at this age. The examination also confirmed a pericardial effusion of about 30 mL and bilateral pleural effusions of a few milliliters.

Pulmonary, hepatic and cardiac tissue fragments collected during autopsy were negative for a panel of viruses tested by PCR. Bacterial culture found postmortem contaminants, i.e., some lactococcus lactis in the liver and lungs, with klebsiella oxytoca after enrichment in the lung tissue.

**Fig. 1:**
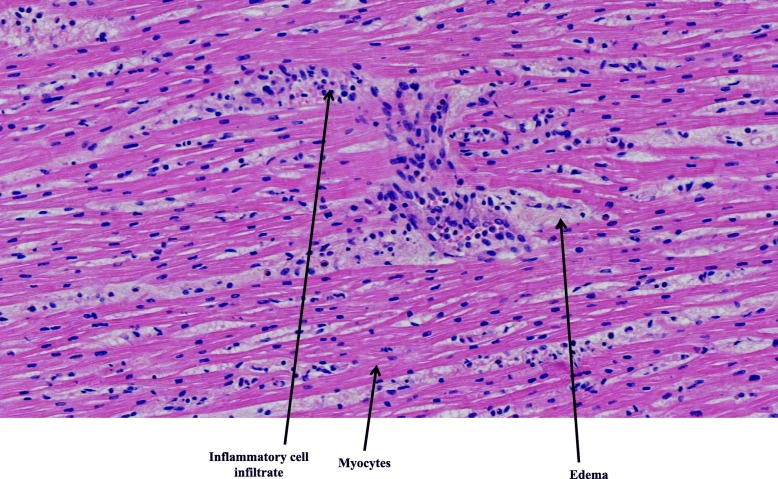
Inflammatory cell infiltrate of the myocardium (lymphocytes and macrophages), with edema and some damaged myocytes. Hematoxylin-eosin staining and magnification of × 20

**Fig. 2:**
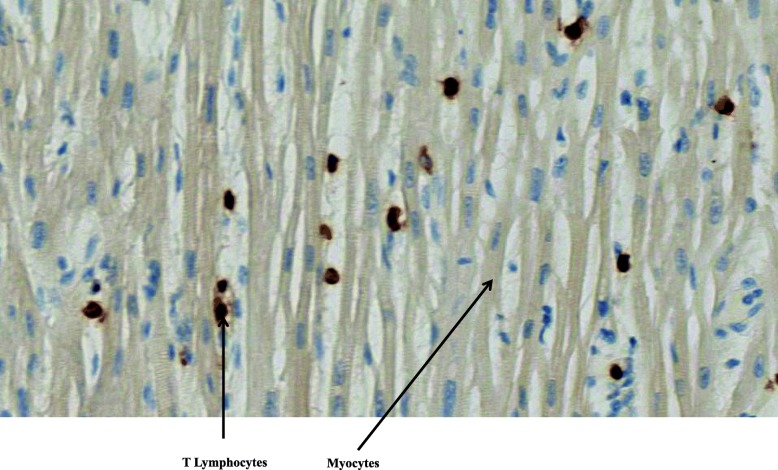
T lymphocytes stained by CD3 in myocardium. Immunohistochemistry and magnification of × 40

**Fig. 3:**
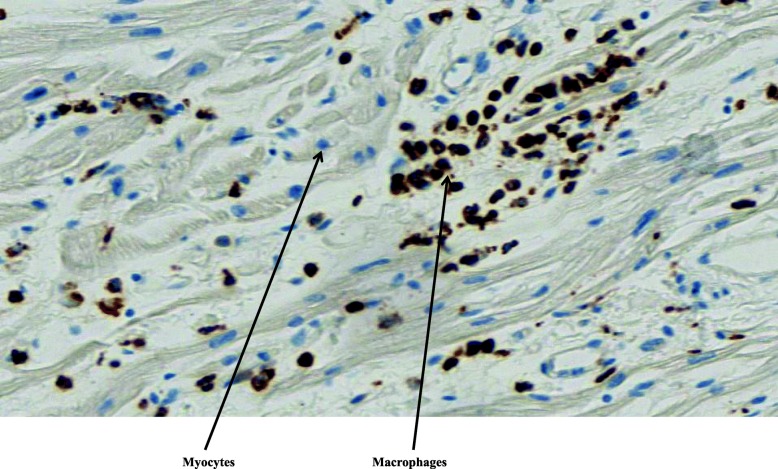
Macrophages stained by CD68 in myocardium. Immunohistochemistry and magnification of × 40

**Fig. 4:**
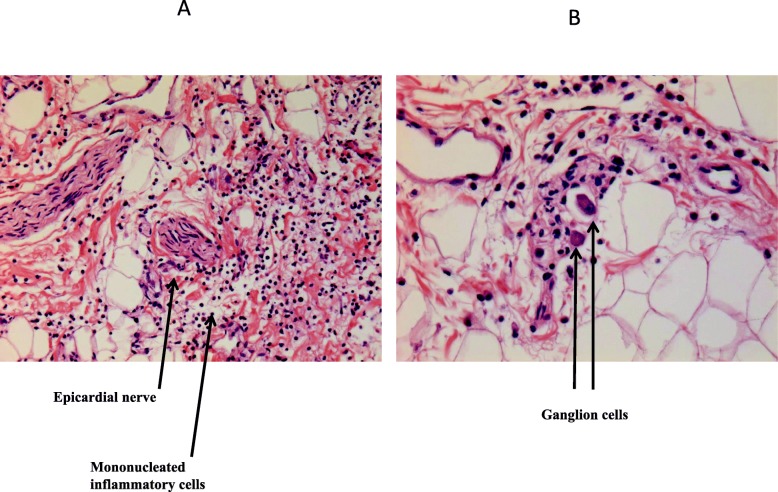
**a** Epicardial nerve surrounded by mononucleated inflammatory cells. Hematoxylin-eosin staining and magnification of × 10. **b** Two ganglion cells surrounded by mononucleated inflammatory cells. Hematoxylin-eosin staining and magnification of × 30

Histopathological examination of the tissues was normal except in the lungs and heart. Diffuse edematous lesions associated with severe alveolar hemorrhages were observed, particularly in the left lung. Rare foci of inflammatory interstitial lesions and discrete bronchitis lesions of the pedicular bronchi were also present in both lungs. Mild to moderate inflammatory infiltrate consisting of T lymphocytes and macrophages (respectively, about 7 per field and 9 per field under × 40 magnification) was found in the myocardium and epicardium, consistent with the diagnosis of myoepicarditis (Fig. 1). Edematous foci and some myocyte changes were visualized. Immunolabeling with anti-CD3, −CD45 and -CD68 antibodies highlighted the inflammatory infiltrates (Figs. 2 and 3). Cardioneuropathy was also observed, with embracing and sometimes penetration of autonomic nerves and ganglion cells by inflammatory cells (Fig. 4).

A joint consultation, in the presence of a pediatrician and psychologist, was held with the parents 7 weeks after the death of their daughter. The parents were told that the most likely cause of death appeared to be a cardiac rhythm disorder occurring in the context of myocardial inflammation. Five months after this first consultation, a new meeting with the family was requested because we had just received the molecular analysis of unexplained sudden death genes on an index case. Among a panel of 31 genes, a heterozygous variation was identified in exon 6 of the myosin light chain 2 (*MYL2*) gene, responsible for the replacement of a glutamic acid by alanine at position 134 p and probably corresponding to a pathogenic variant (class 4 of pathogenicity). At this meeting, we thus proposed a family study to investigate the segregation of this variant with the phenotype and to determine its deleteriousness. Their 20-month-old son was examined by a pediatric cardiologist. Clinical examination was normal, with easily palpated axillary and femoral pulses, normal blood pressure and absence of cardiac murmur. ECG showed a regular sinus rhythm at 126 bpm and conduction and repolarization normal for age. Echocardiography found normal intracardiac architecture, good biventricular function, and the absence of any sign suggestive of cardiomyopathy, valvulopathy or pulmonary arterial hypertension. Molecular analysis of the *MYL2* gene, however, revealed the same heterozygous variation as in his sister. The parents were also examined by an adult cardiologist. ECG and echocardiography were normal for both. The mutation in the *MYL2* gene was observed in the father only.

These elements prompted a new reading of the cardiac histology by two anatomopathologists in two different laboratories, both of whom reported no evidence of arrhythmogenic right ventricular dysplasia or hypertrophic cardiomyopathy (HCM).

## Discussion

We report an exceptional case of sudden death secondary to RSV myoepicarditis, probably complicated by an arrhythmia, in a healthy child of nearly 4 years old.

The diagnosis of myoepicarditis was confirmed histologically and the search for a panel of respiratory viruses by PCR on the pericardial fluid was positive for RSV. The RSV PCR has a specificity greater than 99% [10]. Pericardial effusion or even cardiac tamponade has rarely been associated with RSV infection [11–14]. Effusion may be secondary to CPR, but the fluid is generally bloody because of laceration of the myocardium [15]. Other prolonged chest compression-associated injuries, such as rib fractures and pneumothorax [16], were not observed at autopsy.

We were surprised that no virus was identified in the respiratory samples and tissue fragments collected during autopsy. The lack of a search for bacteriological or viral agents in nasal and throat swabs immediately following death is a limitation of our observation, which may explain why RSV was only found in the pericardial sample. In a study investigating viral infections in cases of SUDI, a considerable variation in the number of RSV diagnoses from lung tissue samples was noted, based on the technique used. All RSV cases were identified with immunohistochemistry, half were positive on real-time PCR, and none on routine shell vial cultures [17].

RSV myocarditis is a well-known extrapulmonary manifestation of severe RSV infection [18]. Although RSV has been detected in human myocardial tissue on PCR [19], the occurrence of cardiogenic shock resulting primarily from heart failure has rarely been documented [20, 21]. Myocardial dysfunction seems a more common clinical picture, notably in infants or in children with congenital heart disease [22–25]. This condition may result from the liberation of inflammatory mediators by infected cells of the respiratory tract, or it may denote the presence of right heart failure due to hypoxia or pulmonary hypertension [26]. Indeed, right ventricular dysfunction has been demonstrated in a minority of patients requiring invasive ventilation [27]. Myocardial injury in these patients might underlie a greater risk for pulmonary complications and cardiovascular deterioration requiring inotropic support [23, 24, 28]. The troponin level has been proposed as a marker of severity and/or an indicator of the contribution of cardiac failure to respiratory distress [23–25]. The level of this protein was not determined in our patient and even if it had been, it could not have been interpreted after prolonged CPR. However, myocardial impairment with mechanical dysfunction is very unlikely in this child who showed no signs of respiratory distress a few hours before her death.

RSV-induced sudden death in normally developed children has been very occasionally reported [29], and the pathogenesis of this event remains to be elucidated. Two fatal cases in 19-month-old toddlers, occurring within half a day of symptom onset, were associated with extensive airway obstruction and remarkably high levels of cytokines IL-6 and IL-8 in the bronchi [30]. On the other hand, ventricular and supraventricular arrhythmias have been described in association with RSV bronchiolitis, including multifocal atrial tachycardia, atrial tachycardia, atrial flutter [31, 32], and ventricular tachycardia with torsades de pointes [12]. In addition, RSV infection may alter the electrical conduction system. Sinoatrial block appears rather common and specific to RSV infection. Its occurrence in infants with mild respiratory symptoms has suggested that RSV might play a direct role in inducing arrhythmia [33]. Complete heart block is a rare complication. It can be contemporary [34] or staggered a few weeks after a documented infection [35]. Endomyocardial biopsy has exceptionally been performed in arrhythmias associated with RSV infections. Sparse lymphocytes and mild perimyocytic fibrosis suggestive of borderline myocarditis were found in a 10-month-old infant with irreversible complete heart block [34]. We speculate that the cardiac nerve lesions observed in our patient were directly involved in her sudden death. James et al. specified in their review on cardioneuropathy that, among possible causes, viral infection and inheritable disorders must be particularly considered [36]. These lesions can indeed cause rhythm disorders by deregulation of the sympathetic/parasympathetic balance. Intracardiac ganglionitis, notably next to the sinus node, was observed in sudden and unexpected deaths in two young and healthy patients. Virus-like particles in the vicinity of the ganglionitis were demonstrated with electron microscopic examination in both cases [37].

The French recommendations in cases of SUDI include genetic testing for long QT syndrome when antecedents in siblings or close ascendants have been documented [2]. We thus identified a heterozygous variation in the *MYL2* gene, a potential pathogenic variant [38]. Glutamic acid at position 134 is highly conserved in evolution and the change to alanine at position 134 p (p.Glu134Ala) was predicted to be pathogenic using a computational tool clinically [39]. In an experimental model of papillary muscle fibers, Burghardt and Sikkink showed that the regulatory light chain harboring the p.Glu134Ala mutation was not able to maintain normal isometric force or normal stiffness, suggesting that actin binding in contraction is compromised by this mutation [40].

This variant in *MYL2* has been reported in a family with HCM [41, 42]. In addition to the affected proband, the variant was identified in two relatives with a clinical diagnosis of HCM and one relative with a phenotype suggestive of HCM (Olivotto I, personal communication). The Laboratory for Molecular Medicine (Partners Health Care Personalized Medicine) in Massachusetts also identified this variant in four individuals with HCM, including two infants who carried a second likely pathogenic *MYL2* variant. In one of these families, each parent had HCM and carried one of the *MYL2* variants, confirming trans-occurrence and suggesting that the combination of the two *MYL2* variants had led to a more severe, early-onset presentation. This combination was found in a Norwegian infant under 1 year with HCM [43]. A case of sudden cardiac arrest was reported in a 32-year-old woman with probable long QT syndrome and the coexistence of variants in *KCNH2* and p.Glu134Ala [44].

In our patient, HCM and arrhythmogenic right ventricular dysplasia were ruled out after careful checking of cardiac histology, and we found no other variant among the unexplained sudden death genes, like *KCNH2*. As the literature shows no report of a rhythm disorder in isolated p.Glu134Ala, we believe that the heterozygous variation in the *MYL2* gene did not play a significant role in the sudden death of this child.

In conclusion, a case of SUDC must mobilize all efforts to clarify the mechanism of death. The investigations performed in this previously healthy 47-month-old girl detected the presence of RSV by PCR in a large pericardial effusion. Postmortem examination played a crucial role in understanding the case, showing myoepicarditis with excessive inflammatory phenomena preferentially affecting cardiac autonomic nerves and ganglion cells, i.e., cardioneuropathy. The fortuitous discovery of a mutation in the *MYL2* gene, which had probably no role in this dramatic event, highlights the complexity of informing parents of a potential genetic cause of death. These conditions are likely to generate unjustified anxiety, especially for siblings whose future is uncertain and who must be subject to prolonged medical follow-up.

## Data Availability

The dataset supporting the conclusions of this article is contained within the manuscript.

## Abbreviations

CPR: Cardiopulmonary resuscitation
CSF: Cerebrospinal fluid
ECG: Electrocardiogram
HCM: Hypertrophic cardiomyopathy
MYL2: Myosin light chain 2
PCR: Polymerase chain reaction
PICU: Pediatric intensive care unit
RSV: Respiratory syncytial virus
SAMU: Urgent medical assistance service
SUDC: Sudden unexpected death in childhood
SUDI: Sudden unexpected death in infancy
